# Retinal Oxygen Kinetics and Hemodynamics in Choroidal Melanoma After Iodine‐125 Plaque Radiotherapy Using a Novel Structural‐Functional Imaging Analysis System

**DOI:** 10.1002/cam4.70854

**Published:** 2025-04-22

**Authors:** Haihan Zhang, Jingyuan Zhu, Yueming Liu, Shiyi Yin, Jinyuan Wang, Yao Yao, Haowen Li, Ximeng Feng, Chuanqing Zhou, Qiushi Ren, Wenbin Wei

**Affiliations:** ^1^ Beijing Tongren Eye Center, Beijing Key Laboratory of Intraocular Tumor Diagnosis and Treatment, Beijing Ophthalmology and Visual Sciences Key Lab, Medical Artificial Intelligence Research and Verification Key Laboratory of the Ministry of Industry and Information Technology, Beijing Tongren Hospital Capital Medical University Beijing China; ^2^ School of Clinical Medicine Tsinghua University Beijing China; ^3^ Department of Biomedical Engineering, College of Future Technology Peking University Beijing China; ^4^ Institute of Biomedical Engineering Shenzhen Bay Laboratory Shenzhen China; ^5^ Institute of Biomedical Engineering Peking University Shenzhen Graduate School Shenzhen China; ^6^ College of Medical Instruments Shanghai University of Medicine and Health Sciences Shanghai China

**Keywords:** choroid melanoma, hemodynamics, plaque radiotherapy, retinal oxygen kinetics

## Abstract

**Background:**

To investigate the changes in retinal oxygen kinetics and hemodynamics in patients with choroidal melanoma (CM) within 2 years before and after iodine‐125 plaque radiotherapy (PRT) using a novel noninvasive structure‐functional imaging analysis system.

**Methods:**

A novel noninvasive cost‐effective imaging analysis system that integrates multimodal structural and functional retinal imaging techniques has been used, which allows rapid acquisition of vascular structural, hemodynamic, and oxygenation metrics using multispectral imaging (MSI) and laser speckle contrast imaging (LSCI) techniques. Follow‐ups have been arranged at the time before plaque implantation surgery, and 1 month, 3 months, 6 months, 12 months, 18 months, and 24 months after iodine‐125 plaque removal.

**Results:**

CM patients after PRT demonstrated a significant decrease in retinal arterial oxygen concentration (CO_2_^a^), arterial oxygen saturation (SO_2_^a^), oxygen utilization (SO_2_^av^, CO_2_^av^), and metabolism (oxygen extraction fraction, OEF) over time. However, there was no significant difference in SO_2_ and CO_2_ compared with healthy controls. Systolic time (Time_sr), acceleration time index (ATI), and resistivity index (RI) gradually increase over time; ATI and RI were significantly higher than those of the healthy controls. At baseline, mean arterial blood flow velocity (BFVa) and mean arterial retinal blood flow (RBFa) in CM eyes were significantly higher than those in the healthy control group. BFVa and RBFa showed a decreasing trend over time after PRT. In addition, some retinal oxygen kinetics and hemodynamic indicators were also correlated with tumor size, patient gender, and age.

**Conclusion:**

CM patients after iodine‐125 plaque radiotherapy had significant retinal and vascular changes. Future research should focus on rapidly screening radiation microvascular complications and exploring more timely and effective interventions to protect visual function in CM patients.

## Graphical abstract

Our study used advanced multimodal imaging techniques to demonstrate significant damage to retinal oxygen metabolism, vascular structure, and retinal blood flow in CM patients after iodine‐125 plaque radiotherapy. These findings may provide new pathophysiological insights into hypoxia in solid tumors after radiotherapy.

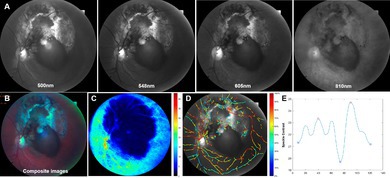

## 1 Introduction

Uveal melanoma (UM) is the most common primary intraocular tumor in adults. It can occur in the iris, ciliary body, and choroid, with choroidal melanoma (CM) accounting for 90% of the cases [1]. The goal of UM therapy is to achieve local tumor control, reduce metastasis risk, and preserve the eyeball and visual function [2]. Treatment selection requires a comprehensive approach, tailored to each UM patient. Plaque radiotherapy (PRT) is the most common eye‐preserving treatment for small–to‐medium‐sized choroidal melanomas (maximum basal diameter ≤ 18 mm, maximum thickness ≤ 12 mm) [3].

Local radiation therapy for many patients diagnosed with UM can control the tumor, preserve the eye, and even preserve some vision, but it often leads to serious radiation complications and vascular damage [4]. Iris neovascularization (4%–23%), neovascular glaucoma (2%–45%), vitreous hemorrhage (4%–18%), and radioactive retinopathy (10%–63%) from ocular vascular damage are common complications after iodine‐125 plaque radiotherapy [5, 6, 7]. Currently, several treatments exist for microvascular complications in UM patients postiodine‐125 plaque radiotherapy, such as photodynamic therapy (PDT), laser photocoagulation, intravitreal dexamethasone and triamcinolone injections, and anti‐VEGF agents [8, 9, 10, 11, 12, 13, 14]. However, research on microvascular changes induced by I‐125 plaque brachytherapy and their correlation with retinal oxygen kinetics and hemodynamics remains limited [15, 16, 17].

Ophthalmic imaging examinations can often detect early retinal diseases before the appearance of visual symptoms, which play an important role in clinical ophthalmology. Due to the hidden onset of early retinal vascular injury after PRT surgery, it is often difficult to identify its clinical features at an early stage using either traditional imaging tools alone, and it needs high requirements for the cooperation of subjects between different tools. Retinal vascular damage can be observed with traditional fundus fluorescein angiography (FFA), which presents retinal vascular leakage, exudative telangiectasia, and capillary dropout [18]. However, FFA is an invasive test, and a small number of patients may even be allergic to the contrast agent. In addition, while structural imaging can help us detect anatomical abnormalities, functional imaging helps to understand the underlying physiological processes, as well as general retinal health and disease. As one of the most metabolically active tissues, the retina consumes oxygen relatively faster than other tissues [19]. In clinical practice, the measurement of retinal oxygen dynamics is essential for understanding the metabolism and pathophysiology of certain eye diseases such as diabetic retinopathy [20] and hypertensive retinopathy [21, 22]. The retinal oxygen saturation (SO_2_), which indicates the percentage of oxygenated hemoglobin in the blood [23], is often used as an important index to estimate retinal oxygen kinetics [24, 25, 26] as well as blood flow velocity (BFV) and lumen diameter (LD) of retinal vessels. In recent years, dual‐wavelength spectral reflection methods have been increasingly used to measure SO2. BFV and retinal vessel diameter can be obtained using techniques like ocular ultrasound Doppler imaging, photoacoustic Doppler velocimetry, and optical coherence tomography angiography (OCTA) [27, 28, 29, 30].

However, few technologies have been reported that can fuse the above structural and functional imaging and provide all parameters simultaneously in a single system. Multimodal integration is a rising research area in biomedical imaging. Integrating multiple functions in a single instrument can reduce costs and simplify clinical work. Therefore, the instrument we used integrates dual‐wavelength imaging and laser speckle contrast imaging (LSCI) technology in the same optical path, proposing a retinal vascular structural and functional imaging and analysis system with high spatial resolution and large field of view (FOV) compared to other devices [31, 32]. The retinal oxygenation results obtained by the traditional dual‐wavelength oxygen saturation method are affected by multiple scattering, changes in retinal reflectance, and pigmentation of the retinal pigment epithelium. Oxygenation values calculated at a single location may have limited precision [33]. To date, most oximetry studies have used similar methods [34]. Our device with four selected wavelengths—500, 548, 605, and 810 nm—enables multispectral imaging (MSI) to highlight retinal vasculature and oxygenation at different retinal layers. LSCI images have physiologically dynamic information, but the contrast and visualization of fine vessels are reduced compared to visible MSI images. The combined data provide complementary information. With the single device, we can obtain and analyze all retinal oxygen metabolism and hemodynamics‐related parameters with high repeatability.

This study employs an innovative multimodal structural–functional imaging system to evaluate vascular structure, hemodynamics, and retinal oxygen kinetics in CM patients after iodine‐125 plaque radiotherapy. By combining and registering multimodal results, we aim to facilitate early detection and monitoring of retinal oxygen and microcirculation changes, advancing both fundamental knowledge and clinical practice.

## 2 Methods

### 2.1 Subjects and Principles

Our study was a nonrandomized, interventional, longitudinal study that included Chinese patients with a clinical diagnosis of CM and treatment with iodine‐125 plaque radiotherapy. At the same time, healthy individuals matched by age and sex were randomly recruited as a control group. Before treatments, the medical history was taken and basic ophthalmic examinations included slit‐lamp biomicroscopy, fundus evaluation, measurements of intraocular pressure (IOP), best‐corrected visual acuity (BCVA), and color Doppler imaging (CDI) to measure the largest basal diameter (LBD) and thickness of the tumor. All subjects completed a comprehensive evaluation before radiotherapy, including blood routine, urine routine, fasting blood glucose, sitting blood pressure, electrocardiogram examination, etc. According to the judgment of the physician, all the indicators of the subjects were in the normal range.

Exclusion criteria were previous interventions in CM, retinal vascular disease (such as diabetic retinopathy, retinal artery occlusion, retinal vein occlusion, hypertensive retinopathy, age‐related macular degeneration [AMD], severe macular scar, or severe subfoveal exudates, etc.), ophthalmic surgery of the affected eye, corneal disease, advanced cataract, and any diseases interfering with the imaging qualities. Peripheral subretinal fluid related to CM was not a criterion for exclusion.

Iodine‐125 plaque radiotherapy was performed using a lead alloy Collaborative Ocular Melanoma Study (COMS)‐type plaque. A radioactively episcleral plaque was placed to cover the entire base of the tumor and at least 2 mm beyond the margin [35]. Amounts of radiative seeds and plaque carrying time were adopted for dosimetric consideration for 100 Gy to the tumor apex. Patients were informed of 2‐year periodic follow‐up after removal of iodine‐125 plaques. The study protocol was approved by the Medical Ethics Committee of the Beijing Tongren Hospital. All subjects were well informed about the study, and written informed consent was obtained.

### 2.2 Imaging Principles and Processing

For all study participants, we performed an integrated retinal imaging instrument (MEFIAS 3200, SYSEYE, Chongqing, China) [31], the principle and methods have been described in previous studies [31]. Limited by the clinical working hours and to avoid the influence of circadian rhythms [36], the examination period for all subjects was between 9 a.m. and 11 a.m. All participants had no intake of drugs influencing haemorheology (10 days preceding the examination). All participants were examined by the same ophthalmologist (HH Zhang). Each participant was asked to fixate on the target within the device and avoid blinking during a 5‐s measuring period for capturing retinal speckle pattern images. During the measurement period, patients were encouraged to keep their breath steady. The device was modified from a regular nonmydriatic fundus camera and integrated with different retinal imaging techniques such as retinal oximetry, laser flowmetry, and oxygen kinetics analysis. Multispectral imaging (MSI) and laser speckle contrast imaging (LSCI) are used for analyses of retinal oxygen kinetics, vascular structure, and hemodynamics.

The patient's medical records were reviewed, and the basic demographic data and past medical history were inquired and recorded to ensure that they did not meet the exclusion criteria. Follow‐up has been arranged at the time before plaque implantation (baseline), and 1 month, 3 months, 6 months, 12 months, 18 months, and 24 months after plaque removal. If a patient developed severe radiation retinopathy during follow‐up and was treated accordingly, such as photodynamic therapy, laser photocoagulation, and intravitreal injection of anti‐vascular endothelial growth factor (VEGF) agents, etc., subsequent follow‐up data from this patient were not included in the analysis.

#### 2.2.1 Applications of MSI Technology

In this study, four wavelengths of 500, 548, 605, and 810 nm were selected for retinal and choroidal illumination and imaging, among which 548 and 605 nm spectral images were used for SO_2_ and oxygen concentration (CO_2_) calculation [31, 34]. One wavelength of 548 nm, which nears the isosbestic absorption point of oxygenated hemoglobin and deoxygenated hemoglobin, is used to observe both arteries and veins. The other wavelength of 605 nm, which is around the maximum difference in the light absorption between the oxygenated and deoxygenated hemoglobin, is sensitive to veins imaging. We selected a peripapillary annulus region of interest (ROI) for retinal SO_2_ estimation. The inner and outer diameters of the annular ROI are 1.5 and 3 times the disc diameter [37]. A trained deep learning model is used to automatically segment blood vessels in dual‐wavelength fundus images.

SO_2_ was often used to estimate retinal oxygenation. SO_2_ changes indicate potential impairments of the retinal blood supply and consequent tissue ischemia or hypoxia [38, 39]. However, analysis of SO_2_ alone is not reliable. Subtle changes in oxygen kinetics, such as oxygen delivery (DO_2_), oxygen metabolism (MO_2_), and oxygen extraction fraction (OEF), can cause serious damage to the retina. Moreover, MSI is also used to measure retinal vascular external diameter (ED) for hemodynamic studies.

#### 2.2.2 Applications of LSCI Technology

Blood flow imaging is another important functional imaging modality. Hemodynamics around the optic disc area is a reliable indicator of blood supply to the retina from the central retinal artery. The LSCI technology quantifies blood flow by measuring the changes in the intensity of speckle pattern images (speckle contrast). When a laser irradiates a rough surface, the resulting high interference leads to a speckle phenomenon. The speckle contrast values are inversely related to blood flow volume (BFV)/minute. The retinal blood flow (RBF) is then estimated by integrating BFV with vessel diameter measurements obtained from MSI. LSCI can also be used to evaluate key pulsatility metrics (Supporting Information S1), including rising rate (RR): the ratio of the area under the systolic curve to the total area of the systolic rectangle in the blood flow pulse curve. The RR reflects the level of blood flow during the systolic phase of the cardiac cycle. Flow acceleration index (FAI) is used to calculate the flow perfusion value with the largest change in the adjacent frame of all the frames in the systolic period. Acceleration time index (ATI) represents the fraction of systole over the entire cardiac cycle. Resistivity Index (RI): the pulse waveform amplitude difference was divided by the maximum value. By combining LSCI‐derived hemodynamic parameters (such as BFV and RBF) with MSI‐derived oxygenation parameters (such as SO_2_ and CO_2_), we can simultaneously achieve structural and functional assessment of the retina.

### 2.3 Statistical Analyses

All statistical analyses were performed using GraphPad Prism 9.0.0 and SPSS 25.0 for paired t‐test or one‐way ANOVA, which were adjusted using Dunnett's Correction to account for multiple comparisons. Variables were presented as mean ± standard deviation or frequency, and the 95% confidence interval (CI) was calculated for continuous variables. In all analyses, *p* < 0.05 (*p < 0.05, **p < 0.01, ***p < 0.001, ****p < 0.0001) was considered to have statistical significance. Pearson correlation, Spearman correlation, and Partial correlation analysis were employed to confirm correlations between retinal oxygen kinetics and hemodynamics with clinical indicators. A *p* value of two‐tailed below 0.05 was thought of as significant.

## 3 Results

### 3.1 The Basic Demographics and Baseline Retinal Oxygen Kinetics and Hemodynamics Parameters

Some patients were lost to follow up after 12 and 18 months (3 and 6 patients, respectively). Finally, a total of 38 patients with CM (38 eyes) who fulfilled the inclusion criteria were included in this study. Among them, 20 [52.63%] patients were men and 18 [47.37%] patients were women, with ages ranging from 12 to 69 years (49.08 ± 11.16 years old), and no one has iris and ciliary body involvement. Twenty‐one [55.26%] patients had the right eye affected, and 17 [44.74%] cases were on the left. The best corrected visual acuity 1.9LogMAR or better, intraocular pressure (IOP) < 22 mmHg, refractive error between −6.00 and +1.50 dioptres, and astigmatism < 1.50 dioptres. The tumor thickness (ranged between 3.46 and 9.5 mm, mean 5.9 mm, SD 1.73) and LBD (ranged between 6.52 and 18.5 mm, mean 12.12 mm, SD 3.35) were measured by the CDI within 2 weeks before plaque implantation.

**FIGURE 1:**
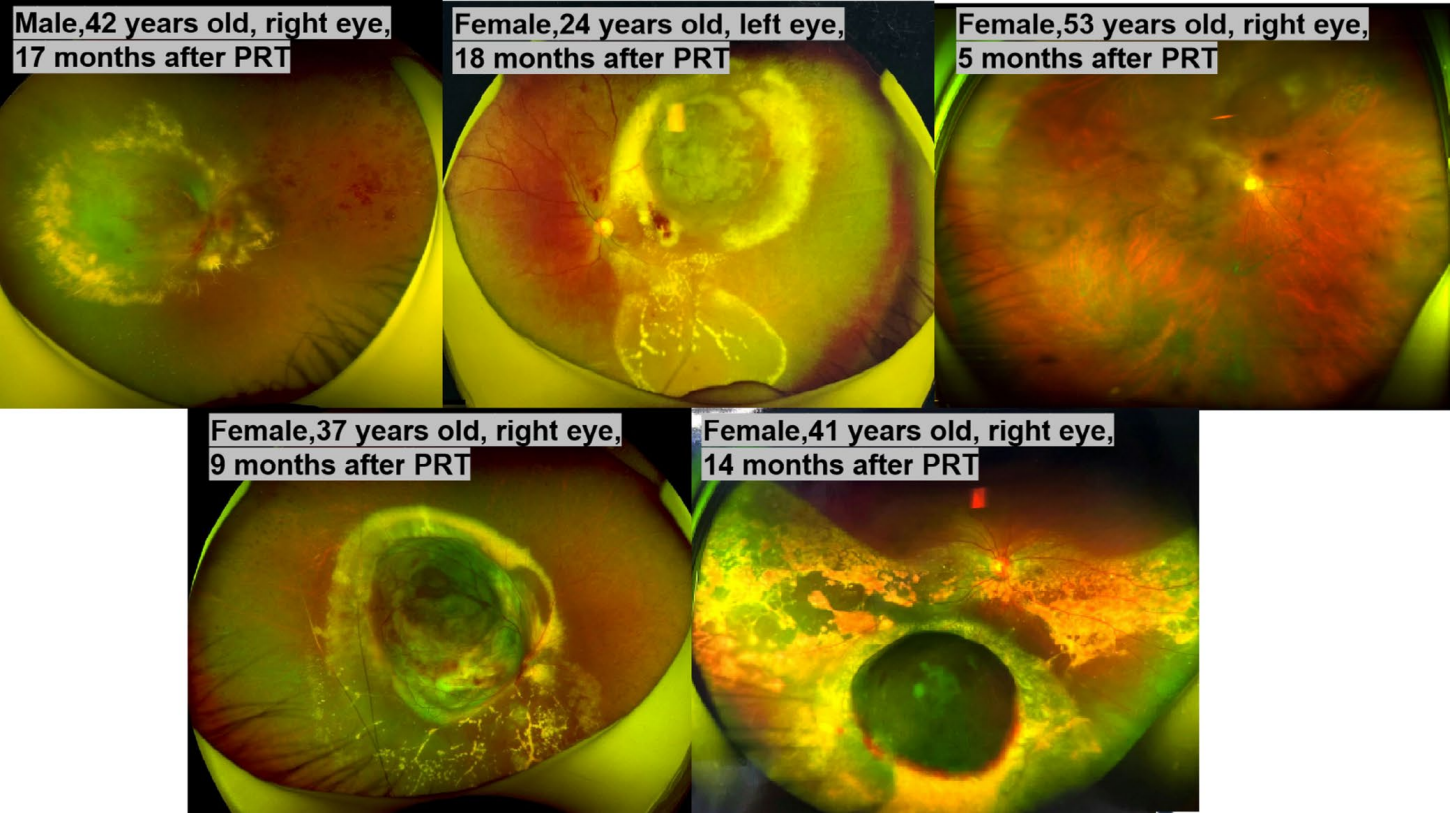
Fundus photographs of five patients who developed radioretinopathy during the follow‐up.

As shown in Figure 1, five patients developed radiation retinopathy during the follow‐up and were treated accordingly. Subsequent follow‐up data were not included in the analysis. Most of these patients have obvious vision decline, and some have metamorphopsia. Fundus photographs showed microaneurysms, retinal hemorrhage, retinal exudation, retinal edema, and retinal or optic disc neovascularization.

**FIGURE 2:**
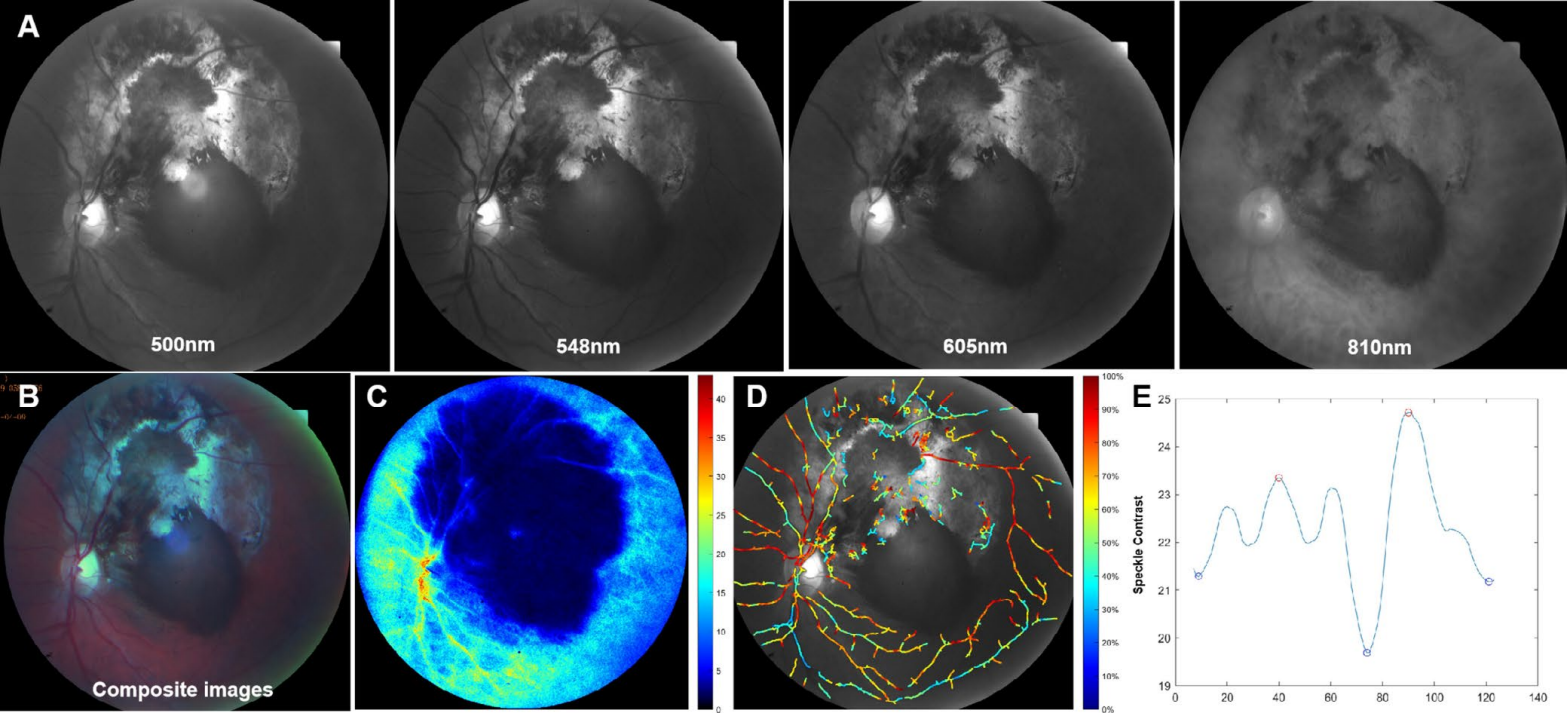
Multimodal retinal blood flow images from CM subject. (A) Multispectral images of 500, 548, 605, and 810 nm. (B) Composite color fundus images of CM eyes. (C) LSCI perfusion image. (D) Retinal SO_2_ mapping in the MSI sequence. (E) Pulsatility waveform generated from LSCI.

Fifty healthy individuals (50 eyes) were recruited as the control group. The CM group and the healthy control group were matched in age (49.08 ± 11.16 vs. 45.92 ± 10.01, *T* = 1.619, *p* = 0.1078) and gender (*χ* ^2^ = 4.000, *p* = 0.136). There was no significant difference in SO_2_ and CO_2_ between the two groups. In the CM group, arterial lumen diameter (LDa) (90.53 ± 39.02 vs. 76.20 ± 15.32, *T* = 2.620, *p* = 0.0097), venous external diameter (EDv) (142.28 ± 24.71 vs. 131.41 ± 21.59, *T* = 2.331, *p* = 0.0210), venous lumen diameter (LDv) (91.99 ± 23.03 vs. 82.08 ± 18.46, *T* = 2.123, *p* = 0.0354), OEF (0.41 ± 0.07 vs. 0.38 ± 0.07, *T* = 2.180, *p* = 0.0308), BFVa (117.35 ± 29.81 vs. 95.49 ± 21.13, *T* = 3.479, *p* = 0.0007), RBFa (62.53 ± 13.91 vs. 51.76 ± 18.50, *T* = 2.173, *p* = 0.0352), and FAI (0.04 ± 0.03 vs. 0.03 ± 0.01, *T* = 3.284, *p* = 0.0013) were significantly higher than those of the healthy control group; the difference was statistically significant (Table 1). A typical multimodal retinal blood flow image of a CM subject is shown in Figure 2.

**TABLE 1:** The baseline data of the CM group and the healthy control group.

Indicators	Unit	CM	Control	*T*/*χ* ^2^	*p*
Age	Year	49.08 ± 11.16	45.92 ± 10.01	1.619	0.1078
Gender	Male/female	20/18	24/26	4.000	0.1360
IOP	mmHg	13.21 ± 3.11	13.80 ± 2.02	1.693	0.0910
SO_2_^a^	%	83.6 ± 5.63	81.03 ± 6.45	1.929	0.0556
SO_2_^v^	%	44.26 ± 7.21	46.25 ± 8.03	1.191	0.2355
CO_2_^a^	mlO_2_/dL	15.93 ± 1.3	15.46 ± 1.43	1.761	0.0802
CO_2_^v^	mlO_2_/dL	8.45 ± 1.45	8.71 ± 1.55	0.826	0.4100
EDa	μm	88.16 ± 19.82	93.5 ± 18.34	1.363	0.1746
LDa	μm	90.53 ± 39.02	76.20 ± 15.32	2.620	0.0097
EDv	μm	142.28 ± 24.71	131.41 ± 21.59	2.331	0.0210
LDv	μm	91.99 ± 23.03	82.08 ± 18.46	2.123	0.0354
BFVa	mm/s	117.35 ± 29.81	95.49 ± 21.13	3.479	0.0007
RBFa	μL/min	62.53 ± 13.91	51.76 ± 18.50	2.173	0.0352
FAI	—	0.04 ± 0.03	0.03 ± 0.01	3.284	0.0013
ATI	—	47.16 ± 14.41	52.1 ± 14.71	1.668	0.0972
RI	—	0.27 ± 0.06	0.31 ± 0.12	1.557	0.1214
OEF	—	0.41 ± 0.07	0.38 ± 0.07	2.180	0.0308

### 3.2 Retinal Oxygenation and Oxygen Kinetics Parameters After Radiation Therapy

**FIGURE 3:**
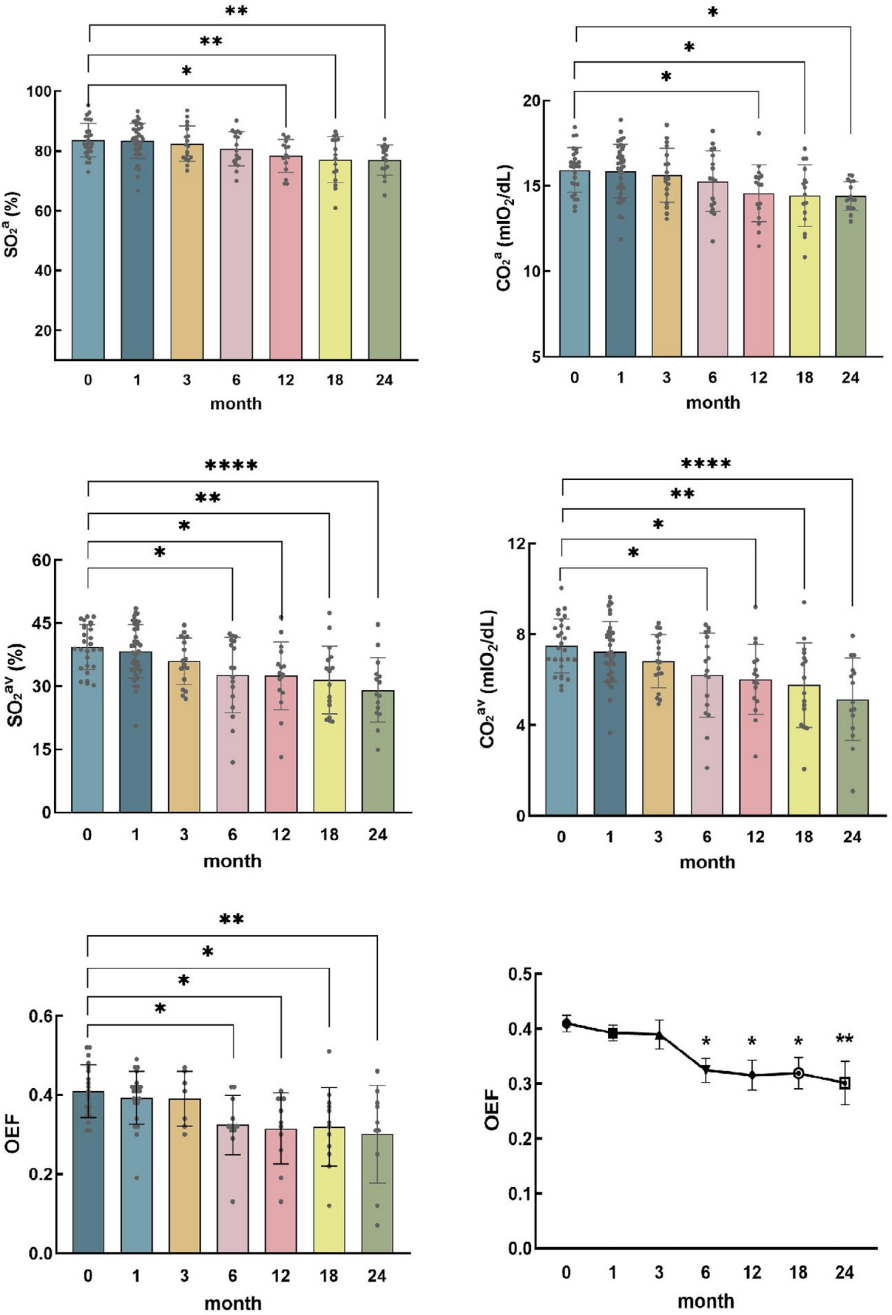
Retinal oxygen saturation and OEF in CM eyes after radiation therapy.

Table 2 shows the parameters of retinal oxygenation and oxygen kinetics at different follow‐up time points in CM eyes after iodine‐125 plaque therapy. The SO_2_^a^, CO_2_^a^, SO_2_^av^, CO_2_^av^ and OEF all decreased gradually after treatment compared with baseline. Starting at 12 months after radiation therapy, SO_2_^a^ and CO_2_^a^ were significantly lower than baseline. Starting at 6 months after radiation therapy, SO_2_^av^, CO_2_^av^ and OEF were significantly lower than baseline (shown in Figure 3, *p < 0.05, **p < 0.01, ****p < 0.0001).

**TABLE 2:** Retinal oxygenation and oxygen kinetics parameters in CM eyes after radiation therapy.

Indicators	Unit	Baseline	1 month	3 months	6 months	12 months	18 months	24 months	*p*
SO_2_^a^	%	83.6 ± 5.63	83.42 ± 5.92	82.47 ± 5.9	80.71 ± 5.72	78.36 ± 5.46	77.16 ± 7.65	77.02 ± 5.13	0.0001
SO_2_^v^	%	44.26 ± 7.21	45.94 ± 6.28	46.52 ± 5.5	48.65 ± 7.35	46.15 ± 9.29	46.94 ± 10.78	51.07 ± 9.48	0.2396
SO_2_^av^	%	39.3 ± 5.35	38.29 ± 5.79	35.95 ± 5.54	32.66 ± 9.01	32.46 ± 8.03	31.47 ± 8.07	29.1 ± 7.66	< 0.0001
CO_2_^a^	mlO2/dL	15.93 ± 1.3	15.87 ± 1.66	15.62 ± 1.58	15.27 ± 1.79	14.58 ± 1.67	14.43 ± 1.8	14.41 ± 0.85	0.0008
CO_2_^v^	mlO2/dL	8.45 ± 1.45	8.65 ± 1.26	8.81 ± 1.19	9.17 ± 1.36	8.57 ± 1.84	8.67 ± 1.84	9.71 ± 1.81	0.2304
CO_2_^av^	mlO2/dL	7.49 ± 1.19	7.22 ± 1.28	6.81 ± 1.17	6.2 ± 1.85	5.81 ± 1.57	5.76 ± 1.86	5.13 ± 1.82	< 0.0001
OEF	—	0.41 ± 0.07	0.39 ± 0.06	0.39 ± 0.07	0.32 ± 0.08	0.32 ± 0.09	0.32 ± 0.1	0.3 ± 0.12	0.0009

### 3.3 Retinal Blood Flow and Vascular Parameters After Radiation Therapy

**FIGURE 4:**
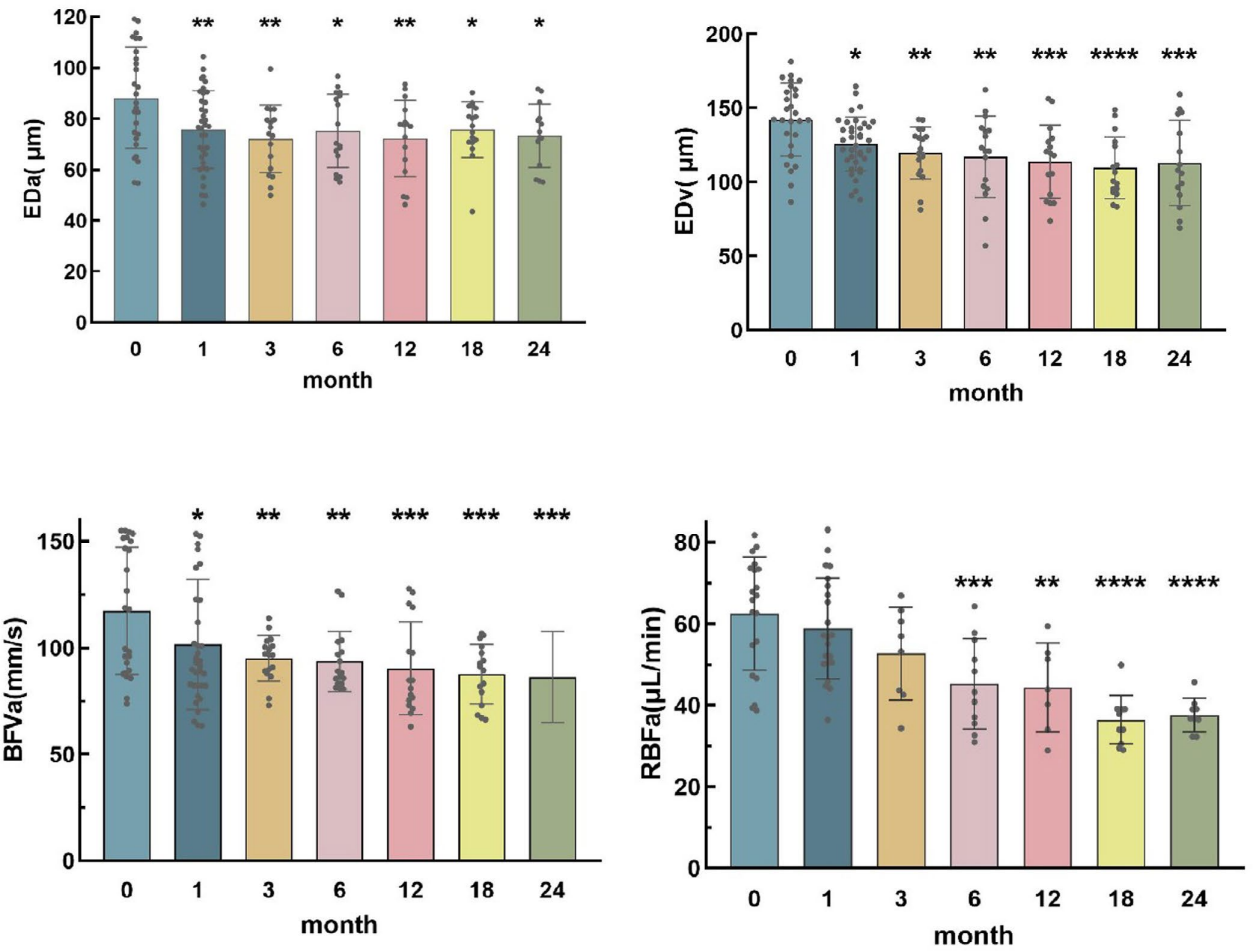
Retinal blood flow and vascular parameters in CM eyes after radiation therapy.

**FIGURE 5:**
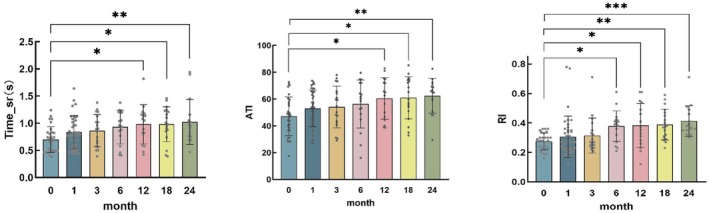
Pulse waveform parameters for ocular circulation in CM eyes after radiation therapy.

As can be seen from Table 3, Figures 4 and 5, EDa and EDv were significantly lower than the baseline data before iodine‐125 plaque therapy over time. WTv increased slightly in the early period and gradually decreased below baseline after 6 months. Compared with the baseline data before iodine‐125 plaque therapy, the BFVa and RBFa in CM eyes decreased significantly over time. As for pulse waveform parameters, systolic time (Time_sr), ATI, and RI in CM eyes were significantly lower than baseline (*p < 0.05, **p < 0.01, ***p < 0.001, ****p < 0.0001).

**TABLE 3:** Retinal blood flow and vascular parameters in UM eyes after radiation therapy.

Indicators	Unit	Baseline	1 month	3 months	6 months	12 months	18 months	24 month	*p*
EDa	μm	88.16 ± 19.82	75.71 ± 16.14	72.14 ± 13.27	75.19 ± 14.38	72.26 ± 14.98	75.76 ± 10.95	73.28 ± 12.46	0.0057
LDa	μm	90.53 ± 39.02	87.9 ± 41.4	99.95 ± 37.36	71.8 ± 32.95	81.31 ± 44.39	100.78 ± 61.16	51.47 ± 21.63	0.1166
WTa	μm	26.47 ± 31.09	29.71 ± 32.29	36.58 ± 28.8	27.19 ± 19.75	23.22 ± 17.59	10.21 ± 7.92	18.56 ± 12.5	0.5155
EDv	μm	142.28 ± 24.71	125.78 ± 16.98	119.76 ± 17.52	117.19 ± 27.49	113.84 ± 24.67	109.67 ± 20.82	112.97 ± 28.79	< 0.0001
LDv	μm	91.99 ± 23.03	88.33 ± 40.23	92.11 ± 34.46	94.46 ± 33.31	96.69 ± 36.13	97.58 ± 39.29	67.08 ± 18.56	0.4314
WTv	μm	26.7 ± 5.29	27.84 ± 12.9	28.22 ± 3.59	24.11 ± 14.22	21.06 ± 9.43	16.72 ± 4.44	17.22 ± 4.8	0.0054
BFVa	mm/s	117.35 ± 29.81	101.65 ± 32.18	95.02 ± 10.74	93.58 ± 14.15	90.45 ± 21.75	87.65 ± 13.96	86.34 ± 21.33	0.0004
RBFa	μL/min	62.53 ± 13.91	58.88 ± 12.12	52.68 ± 11.43	45.27 ± 11.09	44.39 ± 10.88	36.46 ± 5.97	37.62 ± 4.12	< 0.0001
Time_sr	s	0.7 ± 0.24	0.83 ± 0.31	0.86 ± 0.29	0.93 ± 0.31	0.98 ± 0.36	0.98 ± 0.32	1.02 ± 0.42	0.0077
ATI	—	47.16 ± 14.41	52.94 ± 14.04	54.08 ± 15.57	56.29 ± 17.92	60.43 ± 15.5	60.94 ± 15.77	62.4 ± 12.99	0.0083
RI	—	0.27 ± 0.06	0.31 ± 0.15	0.31 ± 0.12	0.38 ± 0.1	0.38 ± 0.15	0.39 ± 0.1	0.41 ± 0.1	0.0002

### 3.4 Parameters of CM Eyes After PRT Compared With the Healthy Control Group

We further compared retinal parameters in CM eyes 2 years after PRT with healthy controls. As shown in Table 4, there was no significant difference in SO_2_ and CO_2_ between the two groups. In the CM group, EDa (73.28 ± 12.46 vs. 93.5 ± 18.34, *T* = 4.038, *p* = 0.0001), LDa (51.47 ± 21.63 vs. 76.20 ± 15.32, *T* = 3.663, *p* = 0.0004), EDv (112.97 ± 28.79 vs. 131.41 ± 21.59, *T* = 3.029, *p* = 0.0029), LDv (67.08 ± 18.56 vs. 82.08 ± 18.46, *T* = 2.479, *p* = 0.0014), and OEF (0.30 ± 0.12 vs. 0.38 ± 0.07, *T* = 3.223, *p* = 0.0016) were lower than those of the healthy control group. Compared to baseline data shown in Table 1, it was evident that LDa, EDv, LDv, and OEF all showed significant declines 2 years after radiotherapy. BFVa and RBFa were significantly increased in the CM eye compared with the control eye before treatment. But BFVa and RBFa decreased gradually after PRT treatment. In particular, RBFa (37.62 ± 4.12 vs. 51.76 ± 18.50, *T* = 2.252, *p* = 0.0311) was significantly lower than that of the healthy control group, and the difference was statistically significant. Furthermore, while no significant differences were noted in ATI and RI between CM eyes and healthy controls at baseline, 24 months after radiation therapy, ATI (62.4 ± 12.99 vs. 52.1 ± 14.71, *T* = 2.677, *p* = 0.0083) and RI (0.41 ± 0.1 vs. 0.31 ± 0.12, *T* = 3.248, *p* = 0.0014) were significantly higher than those of the healthy controls.

**TABLE 4:** Parameters from the CM group at 2 years after PRT were compared with healthy controls.

Indicators	Unit	CM	Control	*T*	*p*
SO_2_^a^	%	77.02 ± 5.13	81.03 ± 6.45	1.929	0.0556
SO_2_^v^	%	51.07 ± 9.48	46.25 ± 8.03	1.191	0.2355
CO_2_^a^	mlO_2_/dL	14.41 ± 0.85	15.46 ± 1.43	1.761	0.0802
CO_2_^v^	mlO_2_/dL	9.71 ± 1.81	8.71 ± 1.55	0.826	0.4100
EDa	μm	73.28 ± 12.46	93.5 ± 18.34	4.038	0.0001
LDa	μm	51.47 ± 21.63	76.20 ± 15.32	3.663	0.0004
EDv	μm	112.97 ± 28.79	131.41 ± 21.59	3.029	0.0029
LDv	μm	67.08 ± 18.56	82.08 ± 18.46	2.479	0.0014
BFVa	mm/s	86.34 ± 21.33	95.49 ± 21.13	1.163	0.2468
RBFa	μL/min	37.62 ± 4.12	51.76 ± 18.50	2.252	0.0311
FAI	—	0.04 ± 0.03	0.03 ± 0.01	1.643	0.1025
ATI	—	62.4 ± 12.99	52.1 ± 14.71	2.677	0.0083
RI	—	0.41 ± 0.1	0.31 ± 0.12	3.248	0.0014
OEF	—	0.30 ± 0.12	0.38 ± 0.07	3.223	0.0016

### 3.5 Correlation Analysis Between Retinal Oxygen Kinetics and Clinical Indicators

**FIGURE 6:**
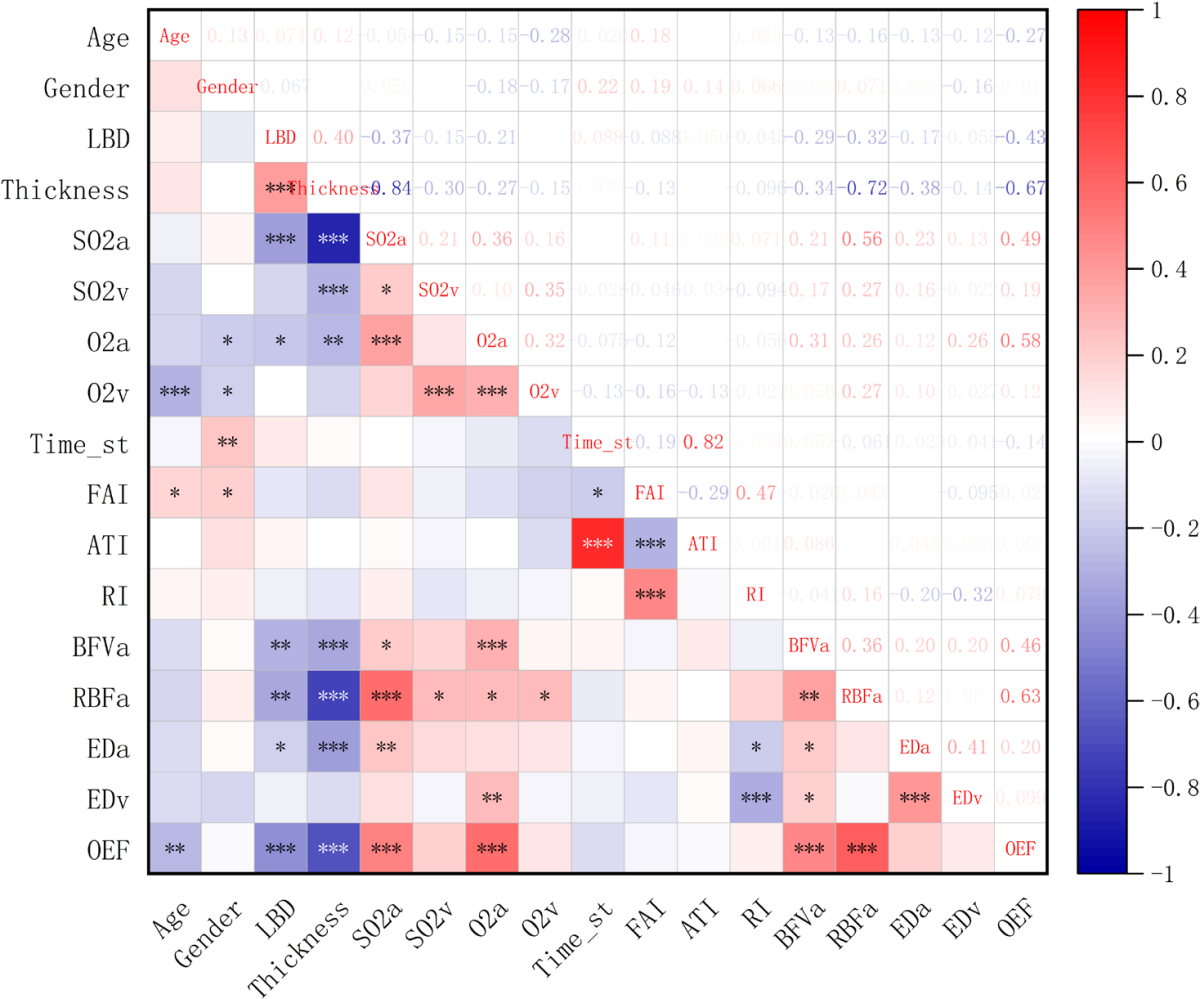
Spearman correlation analysis between retinal oxygen kinetics and clinical indicators (*p value of two‐tailed below 0.05, **p value of two‐tailed below 0.01, ***p value of two‐tailed below 0.001; the *R* values in the figure represent Spearman's correlation coefficients, which range between −1 and 1).

A further comparison has been performed between retinal oxygen kinetics and clinical indicators. As can be seen from Figure 6, the results of the Spearman correlation analysis showed that with elevated tumor LBD, SO_2_^a^, CO_2_^a^, OEF, BFVa, RBFa, and EDa there was a gradually decreasing trend after radiotherapy (correlation coefficient *R* = −0.37, −0.21, −0.43, −0.29, −0.32, −0.17, respectively). With the increase of tumor thickness, SO_2_^a^, SO_2_^v^, CO_2_^a^, OEF, BFVa, RBFa, and EDa showed a gradual decreasing trend (*R* = −0.84, −0.30, −0.27, −0.67, −0.34, −0.72, −0.38, respectively). With the increase of age, CO_2_^v^ and OEF showed a decreasing trend (*R* = −0.28 and −0.27, respectively). As for gender grouping, Time_sr was relatively higher in female subjects (*R* = 0.22).

## 4 Discussion

Retinal structural imaging is a widely used and mature technology in ophthalmic clinical practice. Using tools such as slit‐lamp microscopes, fundus cameras, and optical coherence tomography (OCT), ophthalmologists can assess retinal vascular abnormalities like hemorrhage, exudation, and neovascularization. These assessments aid in diagnosing related diseases. However, as retinal structural imaging has advanced, functional imaging technology has also gained significant attention. Functional imaging provides deeper insights into disease pathogenesis, complementing structural evaluations.

Retinal blood oxygen is a widely studied subclinical indicator of retinal function. Retinal oxygen kinetics can intuitively reflect disease progression. Hypoxia is a hallmark of the tumor microenvironment, promoting cancer progression and therapy resistance. Numerous methods have been developed to measure and evaluate blood oxygen levels of tumors [40, 41]. These include noninvasive imaging methods such as Oxygen‐enhanced MRI and positron emission tomography (PET) using hypoxia tracers and MRI, specifically blood oxygenation level dependent (BOLD) [41, 42]. Oxygen electrodes have been widely used to evaluate oxygenation status in solid tumors [43]. However, this method is invasive, prone to variability, and susceptible to measurement bias based on sensor placement [40, 44]. Despite its advantages, BOLD‐MRI relies heavily on blood flow and does not directly measure tissue oxygenation (pO_2_) or CO_2_ levels [40, 41, 45].

Another oximetry technology, MSI offers the advantage of noncontact and rapid acquisition, presenting through fundus images. It provides both structural information on retinal vasculature and functional data, such as retinal oxygen kinetics. On the other hand, LSCI is a widely used technology for imaging cerebral blood flow, retina, and skin [46]. LSCI offers the advantages of noninvasiveness, high temporal and spatial resolution, rapid data acquisition, and a large FOV. It enables retinal perfusion analysis by providing detailed vascular structure and blood flow information, such as FAI, ATI, RI, RBF, and BFV. Combining MSI and LSCI technologies allows for retinal oxygen kinetics imaging and analysis within a single device. This integration facilitates a comprehensive evaluation of both structural and functional retinal metrics.

Our results show the comparison of retinal oxygenation and oxygen kinetics parameters in CM eyes before and after iodine‐125 plaque radiotherapy. Assessment of oxygen metabolism (MO2) can provide essential information about the cell's ability to generate energy and perform visual processing. There was no significant difference in arterial and venous SO_2_ and CO_2_ between CM eyes and healthy subjects before treatment. After treatment, the SO_2_^a^, CO_2_^a^, SO_2_^av^ and CO_2_^av^ were all significantly lower than the values before treatment in CM eyes, suggesting potential radiation impairments on retinal microvascular oxygen supply in CM eyes, leading to tissue ischemia or hypoxia. Previous studies observed acute hypoxia after radiotherapy in rodent tumors, resulting from a transient closing or blockage of tumor blood vessels, which are often chaotic and malformed [47, 48]. Investigating tumor hypoxia at the clinical level, Kelada et al. demonstrated an increased and persistent state of hypoxia following body radiation therapy to lung cancer patients [49], consistent with our findings. Inadequate oxygen delivery from the abnormal vasculature cannot meet the demands of the rapid proliferation of cancer cells. Hypoxia in solid tumors has become an important predictor of poor clinical prognosis in radiotherapy [50, 51, 52, 53]. Steel et al. discovered that the hypoxic fraction of the tumor varied with its size [54]. In this study, with the increase of tumor thickness and LBD, retinal oxygenation and utilization showed a gradual decreasing trend, which was basically consistent with previous studies.

The OEF is the ratio of oxygen metabolism (MO_2_) to oxygen delivery (DO_2_), quantifying the amount of oxygen that the retinal tissue extracts from the retinal vasculature for metabolism. Referring to the reproducibility of retinal oxygen kinetics indexes in previous studies [31, 32], the OEF values of the healthy subjects included in our study were within the normal range. Compared to the healthy controls, while the OEF of the CM eyes before radiotherapy was significantly higher, it became significantly lower after PRT. In addition, FAI was also higher in CM eyes than in healthy controls. Greater FAI indicates greater systolic pumping capacity. The increase of OEF and FAI may be related to the rapid growth of tumors, vigorous metabolism, and increased tissue oxygen demand. The cellular response to decreased oxygen levels is an attempt to restore homeostasis by improving oxygen delivery, decreasing oxygen consumption, and monitoring tumor cell survival [55]. The results of our study showed that OEF exhibited a decreasing trend from the 6th month after radiotherapy, indicating that the oxygen requirement of tumor tissue decreased after CM was treated with PRT, and tumor growth may be controlled to some extent.

Retinal vascular structure related parameters are critical for oxygen kinetics as well as hemodynamics analysis. Tumor blood vessels are fragile and lack the fundamental architecture of blood vessels in normal tissues [56]. In our study results, LDa, EDv, and LDv in the CM group were significantly higher, and EDa was lower than those in the healthy control group at baseline. In the process of tumor growth, dilated blood vessels provide enough oxygen and nutrients, and remove metabolites to support the growth of the growing tumor. At the same time, tumor cells stimulate the proliferation of vascular endothelial cells by inducing the expression of VEGF, inducing neovascularization and embedding in tumor tissue [49, 54]. While our study showed the reduction in arteriovenous diameter (EDav) and vascular wall thickness (WT), EDa, LDa, EDv, and LDv were significantly lower at 24‐month follow‐up, suggesting vascular atrophy and fibrosis due to radiation injury. It has been reported that high‐dose irradiation‐induced vascular damage causes endothelial cell death, thereby initiating an avalanche of secondary cell death [57]. The death of a small segment of endothelial cells can cause occlusion of a fine blood vessels, preventing nutrient supply and damaging many thousands of tumor cells [58], followed by vascular damage, as well as subsequent degradation of the tumor microenvironment [59]. Structural degeneration of retinal blood vessels became more pronounced after radiation. Irradiated tumor vessels often exhibit reduced endothelial cells, thickened basement membranes, and the formation of collateral circulation. Vascular insufficiency leads to leakage, edema, and lipid exudation. Capillary lumen stenosis and local closure result in ischemia and infarction [35].

These abnormal vessel shapes create geometric resistance, disrupting blood flow. As the WT gradually thins, the vessel's pressure resistance weakens, leading to increased blood flow resistance. In our study, the resistivity index (RI) values in CM eyes postradiation therapy increased significantly over time, indicating stronger peripheral resistance in the circulatory system. Elevated ocular vascular resistance is widely recognized as a key factor in the pathogenesis of retinal vein occlusion [60]. In eyes with malignant tumors, vascular resistance increases due to tumor volume compression. Additionally, blood viscosity rises, and postradiotherapy endothelial damage further elevates vascular resistance. Therefore, a high RI value may serve as a risk predictor for severe radiation retinopathy. However, larger longitudinal studies are needed to validate this prediction. The acceleration time index (ATI) represents the fraction of systole over the entire cardiac cycle. A higher ATI indicates a delayed systolic peak in the waveform (Figure 2). Previous studies have shown a positive correlation of ATI with age and diastolic blood pressure. It would be compatible with an age‐related increase and stiffness in arteries [61]. Aging leads to arteriosclerosis and the reduction of elastic fiber, causing the arterial wall to harden [62]. In our study, the gradual increase in ATI in CM eyes after radiotherapy suggests a depressed retinal vascular buffering capacity. Therefore, the normal blood supply and metabolic requirements of the retina should be affected.

However, there is a lack of technologies and equipment for analyzing retinal microcirculation and oxygen kinetics at the same time. For retinal blood flow measurement, FFA is a clinically recognized technique with a wide field of view (FOV) [63]. It allows direct visualization of retinal microcirculation structure, but the use of contrast media carries potential risks, prolongs imaging time, and may cause discomfort to subjects. When using LSCI and MSI to explore the relationship between hemodynamic parameters and clinical parameters in healthy subjects, it was found that vascular blood flow velocity (BFV) and blood flow volume were significantly negatively correlated with age [62]. This was also seen in our patients.

In our study, BFVa and RBFa were significantly increased in the CM eye compared with the control eye before treatment. It seems that the remodeling of the vascular network can lead to increased BFV, as the body attempts to supply sufficient oxygen and nutrients to the CM eye [64]. However, BFVa and RBFa decreased gradually after PRT treatment. As reported, RBF is affected by many factors, such as blood glucose concentration, blood pressure, and heart rate [65]. The observed decrease in retinal BFV and RBF suggests vascular hypoperfusion postradiation, indicating impaired retinal and choroidal microcirculation. This hypoperfusion may result from capillary blockages, thrombosis, or atherosclerosis caused by radiation therapy, further reducing blood supply. Additionally, pulse waveform parameters may vary between normal eyes and glaucomatous eyes as well as across different stages of glaucoma [66]. No correlation between IOP and pulse waveform parameters was found in our study population.

In this study, we utilized integrated retinal imaging equipment combining LSCI and MSI technologies. This system overcomes the limitations of traditional retinal imaging, enabling comprehensive assessments of retinal oxygen kinetics and hemodynamics. Oxygen dynamic metrics which cover both retinal structure and function were extracted, offering a novel approach for retinal diagnosis and analysis. However, there are several limitations. First, the sample size was relatively small, and the study population was limited to Chinese patients because the recruitment and integration were challenging due to the rarity of the disease. A significant proportion of patients chose primary enucleation at the beginning of the visit because of the disease severity, and a small number of patients were lost to follow up. However, the 2‐year follow‐up and correlation analysis results strongly support our conclusions. Although the device is currently restricted to research use and not commercially available, limiting patient diversity, it has undergone extensive validation in multiple studies [31, 32, 67, 68, 69], demonstrating its accuracy and reliability in measuring retinal oxygenation, blood flow, and hemodynamics. Second, the Blowout score (BOS), Blowout time (BOT), rising rate (RR), and falling rate (FR) reportedly may be used to obtain information on the systemic circulatory status, as a correlation to a quantitative index of ocular circulation [61], but these parameters could not be confirmed as far as the current study. Third, for MSI design, the combination of LED fiber‐based illumination and electronic switching enables rapid image acquisition (< 0.2 s for six images) by eliminating mechanical components. However, severe eye movements can still affect image quality. Additionally, histological validation is lacking for the determination of some subclinical indicators.

In the future, we hope to develop cost‐effective and integrated equipment to facilitate larger studies involving retinal disease patients and those with chronic systemic diseases. The underlying biological mechanisms responsible for the changes in retinal oxygen and blood flow will be explored at the cellular and molecular levels. In summary, to the best of our knowledge, our study is the first to combine MSI and LSCI technologies in CM patients, focusing on retinal vascular structure, oxygen kinetics, blood perfusion, and pulsatility metrics. Our study proves that these oxygen and blood flow kinetic parameters have sufficient sensitivity for detecting the abnormal oxygen metabolism in UM eyes and will certainly be helpful for the diagnosis and early intervention of radiation retinopathy characterized by retinal vascular tissue damage and retinal blood circulation disorders in the future.

## 5 Conclusion

In conclusion, our study used advanced multimodal imaging techniques to demonstrate significant damage to retinal oxygen kinetics, vascular structure, and retinal blood flow in CM patients after iodine‐125 plaque radiotherapy. These findings may provide new pathophysiological insights into hypoxia in solid tumors after radiotherapy. Future studies should aim to rapidly screen patients with radiation microvascular complications by some gold indicators and explore more timely and effective interventions to protect visual function in CM patients.

## Author Contributions

**Haihan Zhang:** conceptualization (equal), data curation (lead), formal analysis (lead), software (lead), writing – original draft (lead), writing – review and editing (lead). **Jingyuan Zhu:** conceptualization (equal), formal analysis (equal), project administration (lead), software (equal), validation (equal), writing – original draft (equal), writing – review and editing (lead). **Yueming Liu:** conceptualization (equal), data curation (equal), project administration (equal), resources (equal), supervision (equal), writing – review and editing (equal). **Shiyi Yin:** data curation (equal), methodology (equal), software (equal), writing – review and editing (equal). **Jinyuan Wang:** conceptualization (equal), data curation (equal), methodology (equal), software (equal), writing – review and editing (equal). **Yao Yao:** data curation (equal), methodology (equal), software (equal), writing – review and editing (equal). **Haowen Li:** data curation (equal), methodology (equal), software (equal), writing – review and editing (equal). **Ximeng Feng:** methodology (equal), project administration (equal), software (equal), writing – review and editing (supporting). **Chuanqing Zhou:** formal analysis (equal), investigation (equal), methodology (equal), project administration (equal), software (equal), writing – review and editing (supporting). **Qiushi Ren:** conceptualization (equal), methodology (equal), project administration (equal), software (equal), writing – review and editing (supporting). **Wenbin Wei:** funding acquisition (equal), methodology (equal), project administration (equal), resources (equal), supervision (equal), writing – review and editing (supporting).

## Ethics Statement

All participants signed the informed consent form. The Medical Ethics Committee of the Beijing Tongren Hospital, Capital Medical University peer‐reviewed and approved the related documents and all study protocols for the collection and use of participants' data. The committee's reference number is TRECKY2018‐056. The research followed the tenets of the Declaration of Helsinki. All information is kept confidential, and the patients' basic information will not be made available to other nonresearchers or organizations.

## Conflicts of Interest

The authors declare no conflicts of interest.

## Supporting information


Data S1:

Figure S1:

## Data Availability

The data underlying this article are available in the article and in its online Supporting Information S1 and Figure S1.
