# Supplementary material for: Retinal Oxygen Kinetics and Hemodynamics in Choroidal Melanoma After Iodine‐125 Plaque Radiotherapy Using a Novel Structural‐Functional Imaging Analysis System
Source: Cancer Med. 2025 Apr 22;14(8):e70854. doi: 10.1002/cam4.70854 (PMC12012311; doi:10.1002/cam4.70854)
Supplement: Supplementary file 1 — Data S1: [file CAM4-14-e70854-s001.docx]

LSCI can also be used to evaluate key pulsatility metrics, including:

Rising rate (RR): The ratio of the area under the systolic curve to the total area of the systolic rectangle in the blood flow pulse curve. As shown in Figure 1(a). The RR reflects the level of blood flow during the systolic phase of the cardiac cycle.

Flow acceleration index(FAI)：FAI is used to calculate the flow perfusion value with the largest change in the adjacent frame of all the frames in the systolic period. As shown in Figure 1(b). A larger FAI indicates greater systolic pumping capacity.

Acceleration time index (ATI): represents the fraction of systole over the entire cardiac cycle. As shown in Figure 1(c).

Resistivity Index (RI): the pulse waveform amplitude difference was divided by the maximum value. As shown in Figure 1(d). A larger RI value means stronger peripheral resistance.

By combining LSCI-derived hemodynamic parameters (such as BFV and RBF) with MSI-derived oxygenation parameters (such as SO_2_ and CO_2_), we can simultaneously achieve structural and functional assessment of the retina.


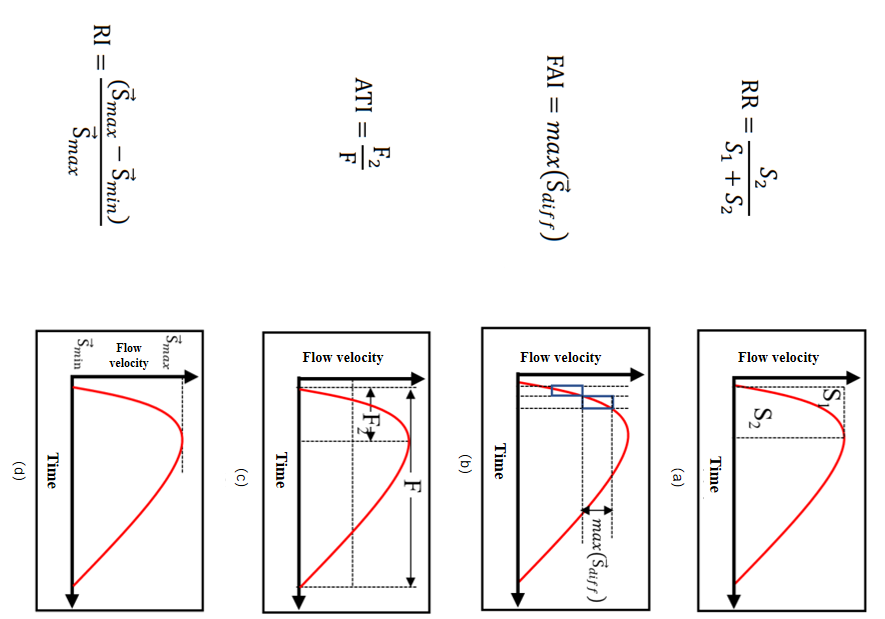


**Supplementary Figure 1. Description of calculation of retinal blood pulsatility metrics.**

S_1_ represents the area on the systolic curve, S_2_ represents area under systolic curve;

F represents the duration of complete cardiac cycle, F_2_ represents the duration of systole;

S_diff_ represents a frame-by-frame differential signal for blood flow pulsation;

S_max_ indicates the signal peak value, and S_min_ indicates the signal valley value.
