# Supplementary figures and images for: Retinal Oxygen Kinetics and Hemodynamics in Choroidal Melanoma After Iodine‐125 Plaque Radiotherapy Using a Novel Structural‐Functional Imaging Analysis System
Source: Cancer Med. 2025 Apr 22;14(8):e70854. doi: 10.1002/cam4.70854 (PMC12012311; doi:10.1002/cam4.70854)

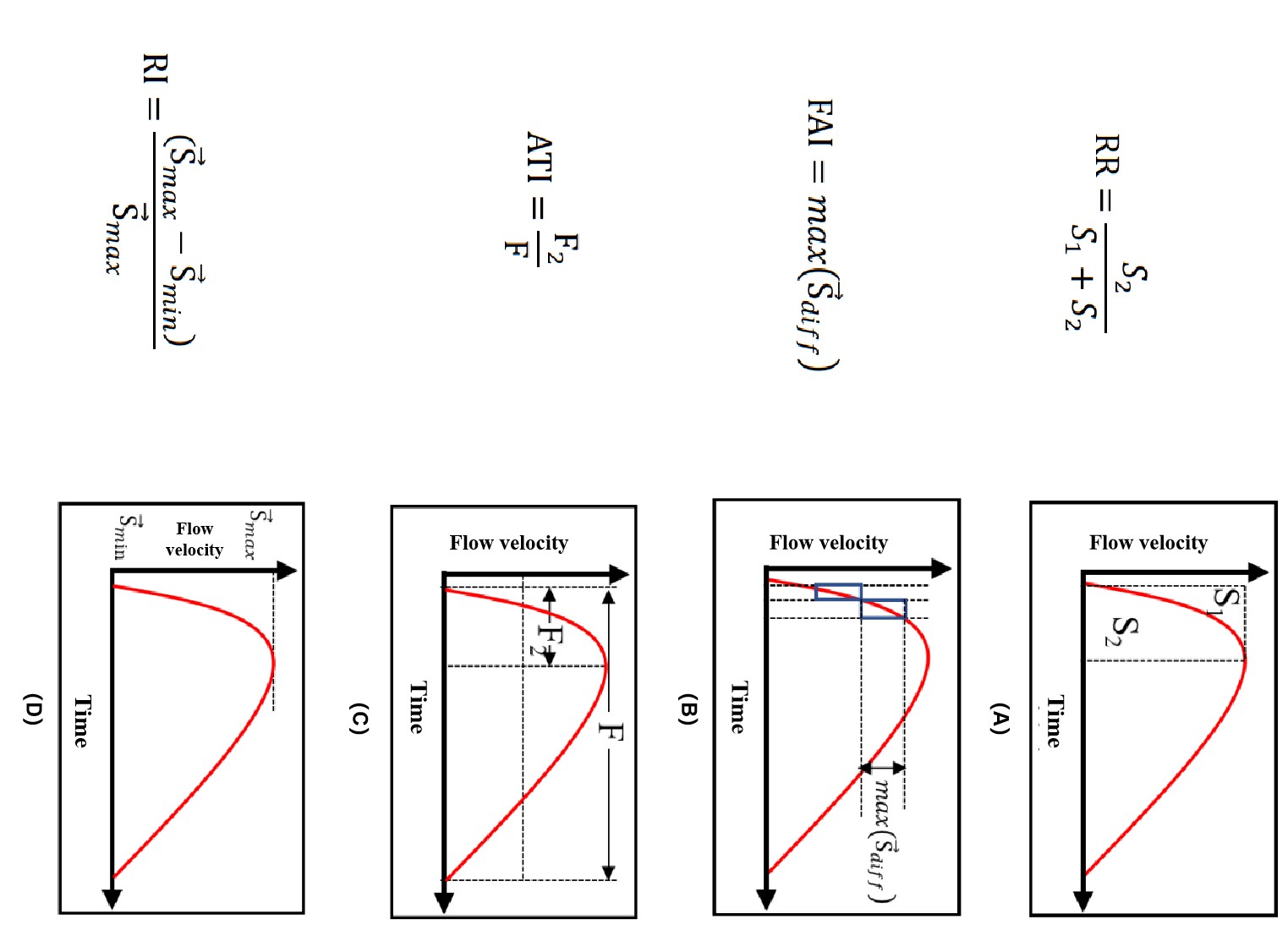

Supplement: Supplementary file 2 — Figure S1: [file CAM4-14-e70854-s002.jpg]
